# Rapid point of care testing for four bacterial sexually transmitted infections using the portable isothermal loop-mediated nucleic acid amplification eazyplex platform

**DOI:** 10.1007/s15010-023-01981-5

**Published:** 2023-01-14

**Authors:** Ece T. Esitgen Germaner, Lars Wassill, Karl Dichtl, Julia Roider, Ulrich Seybold

**Affiliations:** 1 Sektion Klinische Infektiologie, Med. Klinik und Poliklinik IV, LMU Klinikum Innenstadt, Ludwig-Maximilians-Universität München, Pettenkoferstr. 8a, 80336 Munich, Germany; 2 AmplexDiagnostics GmbH, Werkstr. 2, 83555 Gars-Bahnhof, Germany; 3 Max von Pettenkofer-Institut für Hygiene und Medizinische Mikrobiologie, Medizinische Fakultät, Ludwig-Maximilians-Universität München, Pettenkoferstraße 9a, 80336 Munich, Germany; 4 Diagnostik & Forschungsinstitut für Hygiene, Mikrobiologie und Umweltmedizin, Medizinische Universität Graz, Neue Stiftingtalstraße 6/III, 8010 Graz, Austria; 5 ﻿German Center for Infection Research (DZIF), Partner Site Munich, Munich, Germany

**Keywords:** Bacterial sexually transmitted infection, Nucleic acid amplification, Point-of-care-test, People living with HIV, Men who have sex with men—MSM

## Abstract

**Purpose:**

To analyze sensitivity and specificity of the rapid point-of-care (POC) eazyplex testing platform for bacterial sexually transmitted infections (STI) among men who have sex with men (MSM).

**Methods:**

272 anal, urethral, and pharyngeal swabs collected from 153 MSM were tested by both the eazyplex platform and an in-house PCR or culture in the university microbiology reference laboratory.

**Results:**

Compared to the reference diagnostic method, the overall sensitivity/specificity of eazyplex was 88%/98% for *N. gonorrhoeae*, 82%/100% for *C. trachomatis*, 70%/ > 99% for *U. urealyticum,* and 85%/98% for *M. hominis*, respectively. Sensitivity for *N. gonorrhoeae* and *U. urealyticum* in urethral samples was 100%.

**Conclusion:**

With good to very good sensitivity depending on the sampling site and pathogen as well as very good specificity overall the eazyplex platform is a useful rapid diagnostic method for POC bacterial STI-testing especially for *N. gonorrhoeae* and *C. trachomatis*, allowing for almost immediate treatment initiation.

## Introduction

In 2020 the World Health Organization estimated the worldwide number of new cases of infections by *Chlamydia trachomatis* at 129 million and by *Neisseria gonorrhoeae* at 82 million [1]. If not treated rapidly and adequately, sexually transmitted infections (STI) can lead to a series of secondary diseases and complications, such as pelvic inflammatory disease, ectopic pregnancies, infertility, preterm delivery, increased prenatal and postnatal mortality, and cervical cancer [1, 2]. Moreover, concurrent STI enhance HIV transmission [3]. As a result, STI pose a major public health problem worldwide. The key issues to be addressed are rapid diagnosis followed by counseling and therapy.

Nucleic acid amplification testing (NAT) and/or microbiological culture (especially for *N.* *gonorrhoeae*) have traditionally been diagnostic methods of choice for STI. Both usually rely on a central laboratory leading to delays due to sample transport and reporting of results. This requires a follow-up appointment and patients who test positive but do not return will not be treated [4].

Point-of-care- (POC-) NAT also offers the opportunity of testing for STI. The brief report time allows for immediate initiation of treatment and counseling, which are fundamental to breaking the chain of infection. The eazyplex platform (AmplexDiagnostics, Gars-Bahnhof, Germany) tests two samples for up to six bacterial STI pathogens simultaneously. The result is available in approximately 30 min. The objective of this study was to analyze the sensitivity and specificity of this test system for relevant STI among men who have sex with men (MSM) in Germany compared to standard diagnostic tests performed in a reference microbiology laboratory.

## Patients and methods

This single-center study was performed at the Infectious Diseases outpatient clinic of LMU University Hospital in Munich, Germany. Consecutive patients were asked to participate between 27 Nov 2018 and 3 Aug 2020. STI testing was performed upon clinically suspected infection, following specific high-risk contacts, as well as for routine surveillance. Swab samples were taken from the pharynx, urethra, and/or anus using the eSwab system (Copan S.P.A, Brescia, Italy). The standard tip was used for pharyngeal and anal specimens and the mini tip for urethral specimens. After swab collection 100 µl of the modified Amies transport medium were transferred into an additional sterile tube, leaving 900 µl in the eSwab tube.

The eazyplex system is based on isothermal loop-mediated amplification (LAMP) of nucleic acids [5, 6]. It is suited for the detection of six bacterial STI-causing pathogens: *C.* *trachomatis*, *N.* *gonorrhoeae*, *Ureaplasma urealyticum*, *Mycoplasma hominis*, *Mycoplasma genitalium*, and *Treponema pallidum*. The test is conducted in a small, portable, and battery-operated device. During the process, six different primers are used to detect eight specific areas of the target gene. The reaction occurs at a constant temperature of 66 °C and denaturation of the double-stranded DNA into single-stranded DNA is not required. Amplification is measured by real-time fluorescence detection using a commercially available DNA intercalating dye. Data interpretation is automatically performed by the integrated software (Amplex Diagnostics). Results are reported as positive in real-time if the fluorescence level and the peak of the first derivative of the fluorescence curve rise above the defined thresholds in the respective tube of the strip.

From the collection tube, 25 µl of transport medium are removed and transferred to 500 µl of the resuspension and lysis fluid (RALF) buffer, which are incubated in a heating block for 2 ml tubes at 99 °C for 2 min. Subsequently, 25 µl of RALF lysate are transferred to each of the wells of the test strips containing the freeze-dried reagents and the amplification process is started. Final result readings are available after 30 min.

The first batch of 124 samples was tested at the AmplexDiagnostics site. The next 148 samples were analyzed as point-of-care tests at the outpatient clinic by medical staff. For both study periods reference testing was performed at the University of Munich routine clinical microbiology laboratory using the remaining 900 µl from the eSwab tube. *N. gonorrhoeae* was cultured on Thayer-Martin agar and identified by MALDI-TOF analysis. NAT targeting *C. trachomatis* (cryptic plasmid)*, M. hominis* (GAPDH gene), and *Ureaplasma* (urease complex locus) was performed using an *in-house* PCR via the BDmax system (Becton, Dickinson and Company, Franklin Lakes, USA). At the time, only NAT for *Ureaplasma* spp. without further differentiation and NAT for *M. hominis* but not *M. genitalium* was offered by the reference laboratory. In addition, a *Treponema pallidum* particle agglutination (TPPA) based serology was performed using a serum sample (Serodia TPPA, Fujirebio, Tokyo, Japan). In case of positive findings in routine diagnostics, therapy was initiated according to current guidelines [7].

## Results

**Fig. 1:**
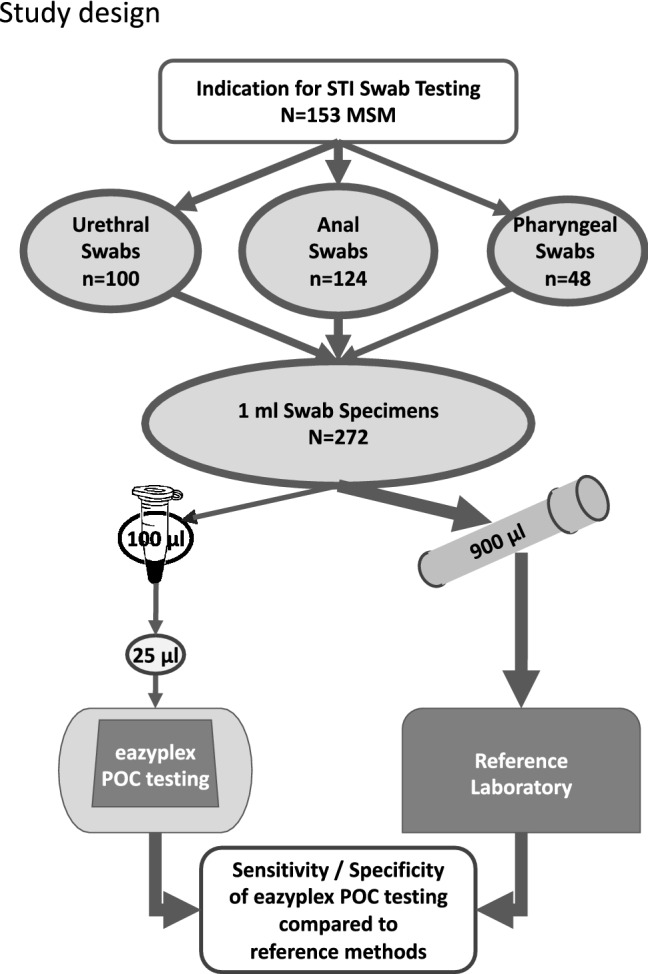
Study design. Note: MSM, men who have sex with men; POC, point-of-care

For this study 272 samples were collected from 153 participants, 144 (94%) of whom were people living with HIV. All participants were MSM, median age was 46 years (range 21–73). Of these 272 samples, 124 were collected from the anus, 100 from the urethra, and 48 from the pharynx (Fig. 1).

Compared to the corresponding routine diagnostic method conducted at the reference clinical microbiology laboratory, the overall sensitivity/specificity of the eazyplex platform was 88%/98% for *N. gonorrhoeae*, 82%/100% for *C. trachomatis*, 70%/ > 99% for *U. urealyticum,* and 85%/98% for *M. hominis*, respectively (Table 1).

**Table 1:** Sensitivity and specificity of point-of-care testing for sexually transmitted bacteria using the eazyplex platform compared to routine testing by the reference clinical microbiology laboratory

Pathogen	Number of samples tested^a^	Reference method	Sensitivity^c^	Specificity
Urethral samples only (*n* = 100)				
*N. gonorrhoeae*	99	Culture	13/13 (100%)	83/86 (97%)
*C. trachomatis*	86	PCR	8/10 (80%)	76/76 (100%)
*U. urealyticum*	99	PCR^b^	14/14 (100%)	85/85 (100%)
*M. hominis*	99	PCR	5/6 (83%)	92/93 (99%)
Anal samples only (*n* = 124)				
*N. gonorrhoeae*	122	Culture	9/12 (75%)	109/110 (99%)
*C. trachomatis*	122	PCR	15/17 (88%)	105/105 (100%)
*U. urealyticum*	124	PCR^b^	19/31 (61%)	92/93 (99%)
*M. hominis*	124	PCR	18/21 (86%)	100/103 (97%)
Pharyngeal samples only (*n* = 48)				
*N. gonorrhoeae*	48	Culture	n/a	48/48 (100%)
*C. trachomatis*	47	PCR	0/1	46/46 (100%)
*U. urealyticum*	48	PCR^b^	0/2	46/46 (100%)
*M. hominis*	48	PCR	n/a	46/48 (96%)
All sampling sites (*N* = 272: pharynx *n* = 48, urethra *n* = 100, anus *n* = 124)				
*N. gonorrhoeae*	269	Culture	22/25 (88%)	240/244 (98%)
*C. trachomatis*	255	PCR	23/28 (82%)^d^	227/227 (100%)
*U. urealyticum*	271	PCR^b^	33/47 (70%)	223/224 (> 99%)
*M. hominis*	271	PCR	23/27 (85%)^e^	238/244 (98%)

*PCR* polymerase chain reaction

^a^Results from the reference laboratory were not available for all POC test results, resulting in fewer paired tests used for the calculation of test characteristics

^b^PCR detected *Ureaplasma spp.* without differentiation between *U. urealyticum* and *U. parvum*

^c^Calculation of sensitivity was not performed for pharyngeal samples due to absent or very rare positive results from the reference laboratory

^d^In 3/5 false negative samples bacterial load was low (cycle threshold value ≥ 30 in PCR)

^e^In 2/4 false negative samples bacterial load was low (cycle threshold value ≥ 30 in PCR)

In samples collected from the anus only, the eazyplex platform showed a sensitivity/specificity of 75%/99% for *N. gonorrhea*, 88%/100% for *C. trachomatis*, 61%/99% for *U. urealyticum* and 86%/97% for *M. hominis*. In urethral samples only, sensitivity/specificity for the eazyplex platform were 100%/97% for *N. gonorrhoeae*, 80%/100% for *C. trachomatis*, 100/100% for *U. urealyticum*, and 83%/99% for *M. hominis*.

Calculation of sensitivity for pharyngeal samples was not possible due to the scarcity of positive results by the respective reference method with 0/48 positive for *N. gonorrhoeaea,* 1/47 for *C. trachomatis*, 2/48 for *U. urealyticum,* and 0/46 for *M. hominis*. The rare positive pharyngeal results for *C. trachomatis* and *U. urealyticum* however remained undetected by the eazyplex platform. Specificity for pharyngeal samples was 100% for *N. gonorrhoeae*, *C. trachomatis*, and *U. urealyticum*, as well as 96% for *M. hominis*.

*T. pallidum* was not detected by the eazyplex platform in any sample, 2/272 were inconclusive/invalid. Serology was compatible with current syphilis infection in 10 cases. Since swabs were not collected from specific lesions and no other direct test was available for comparison sensitivity/specificity were not calculated for *T. pallidum.*

When restricting the calculation to the 261 samples collected from only the 144 people living with HIV the test characteristics for the eazyplex platform remained essentially unchanged (data not shown).

## Discussion

This study showed that POC-testing using the eazyplex platform resulted in good sensitivity for *N. gonorrhoeae* (75–100%), *C. trachomatis* (77–88%), as well as *M. hominis* (83–86%) and very good specificity (≥ 97%) compared to the reference laboratory-based diagnostic methods.

In our study sensitivity depended on sample site and pathogen, whereas specificity was very good overall. Sensitivity for *C. trachomatis* was similar in urethral and anal samples (80% and 88%, respectively), in 3/5 false negative samples bacterial load was low with cycle threshold values of ≥ 30 by the reference PCR method. For *N. gonorrhoeae* sensitivity was higher among urethral than anal samples (100% vs. 75%). Likewise, sensitivity for detection of *U. urealyticum* was very good for urethral samples, but only modest for anal samples (100% vs. 61%). However, in this analysis the reference PCR method at the time did not differentiate between *Ureaplasma* spp., therefore, the potential presence of *U. parvum* would have led to an underestimation of sensitivity for *U. urealyticum*. A higher frequency of *U. parvum* on the anal mucosa compared to the urethra could therefore be responsible for at least part of the observed difference. There were too few positive pharyngeal samples to perform meaningful calculations with respect to sensitivity for this bodysite.

Novel POC test systems using loop-mediated amplification have increasingly been introduced in the last years for different infectious agents, e.g. for malaria in pregnant women [8], SARS-CoV-2 [9], or *C. trachomatis* [10]. With a rising incidence of STI in selected populations the need for POC tests has increased for this indication with accuracy and reasonable pricing being the main factors required for implementation [11]. Already in 1999, a study demonstrated that a rapid test for *C. trachomatis* with a modest sensitivity of 63% resulted in the treatment of more infected patients than following conventional PCR testing, if the proportion of patients returning was less than 65% [12]. Thus, POC rapid testing can be a good alternative in situations when patients may not return to the clinic for a second visit. Being able to receive appropriate treatment almost immediately after diagnostic sampling was considered a valuable advantage by the majority of participants during the second phase of our study, both with respect to symptom relief and protection of contact persons. An immanent limitation of many POC platforms including this eazyplex STI-panel is the inability to detect bacterial drug-resistance, in which case guidelines for primary treatment recommendations may not be appropriate. However, this also is true for the PCR-based testing currently offered by our reference laboratory. In addition, drug-resistance resulting in treatment failure is still infrequent in our setting with the notable exception of *M. genitalium*, and none of the *N. gonorrhoeae*-isolates in this study were resistant to ceftriaxone.

In addition to rapid results within about 30 min as well as good sensitivity and very good specificity the eazyplex platform also meets other ASSURED criteria (Affordable, Sensitivity, Specificity, User friendly, Rapid, Equipment free, Delivered) [13] developed by the World Health Organization, qualifying it for use even in resource-limited settings: it is user-friendly allowing for operation after minimal training, equipment-free as a stand-alone platform, robust with no need for refrigeration, and as a small, portable device can be easily delivered to the end user. While it does not cover the vast range of sexually transmissible pathogens, at our site the overwhelming majority of STI requiring treatment is caused by *C. trachomatis*, *N. gonorrhoeae*, *U. urealyticum*, *M. genitalium*, and *T. pallidum*. The eazyplex platform, therefore, detects the pathogens most relevant for urethritis and proctitis while the short time to result allows for further diagnostic work-up in case of a negative reading.

An obvious limitation of our study was the single-center setting. However, the baseline characteristics of the study participants and testing indications correspond well with the typical setting of MSM outpatient STI testing in Germany. In addition, the number of samples allowed for robust analysis of sensitivity and specificity for urethral and anal sampling sites. Reference laboratory testing at the time somewhat limits our conclusions to mainly *N. gonorrhoeae* and *C. trachomatis*. However, even the potentially underestimated sensitivity for *U. urealyticum* in anal samples is still in the range of clinical usefulness compared to routine testing requiring a follow-up appointment. While the eazyplex platform included testing for *M. genitalium*, again reference testing at the time only allowed for the calculation of test characteristics for *M. hominis*, which is less clinically relevant. If results for *M. hominis* were used as an indication for test characteristics with respect to *M. genitalium*, clinical usefulness can be assumed for this pathogen also.

In conclusion, our study demonstrated that the eazyplex platform is a useful rapid diagnostic method for POC bacterial STI testing, especially for *N. gonorrhoeae* and *C. trachomatis.* In addition to good sensitivity and very good specificity it offers the clinical benefit of almost immediate treatment initiation.

## Data Availability

The data that support the findings of this study are available from the corresponding author, U.S., upon reasonable request.
